# Rice Stomatal Mega-Papillae Restrict Water Loss and Pathogen Entry

**DOI:** 10.3389/fpls.2021.677839

**Published:** 2021-06-04

**Authors:** Mutiara K. Pitaloka, Emily L. Harrison, Christopher Hepworth, Samart Wanchana, Theerayut Toojinda, Watchara Phetluan, Robert A. Brench, Supatthra Narawatthana, Apichart Vanavichit, Julie E. Gray, Robert S. Caine, Siwaret Arikit

**Affiliations:** ^1^Faculty of Agriculture at Kamphaeng Saen, Kasetsart University, Nakhon Pathom, Thailand; ^2^Department of Molecular Biology and Biotechnology, University of Sheffield, Sheffield, United Kingdom; ^3^Department of Animal and Plant Sciences, University of Sheffield, Sheffield, United Kingdom; ^4^National Center for Genetic Engineering and Biotechnology (BIOTEC), National Science and Technology Development Agency (NSTDA), Khlong Luang, Thailand; ^5^Center for Agricultural Biotechnology, Kasetsart University, Kamphaeng Saen Campus, Nakhon Pathom, Thailand; ^6^Thailand Rice Science Institute, Rice Department, Ministry of Agriculture and Cooperatives (MOAC), Suphanburi, Thailand; ^7^Rice Science Center, Kasetsart University, Nakhon Pathom, Thailand; ^8^Department of Agronomy, Faculty of Agriculture at Kamphaeng Saen, Kasetsart University Kamphaeng Saen Campus, Nakhon Pathom, Thailand

**Keywords:** stomata, subsidiary cells, papillae, silicon, gas-exchange, bacterial pathogen

## Abstract

Rice (*Oryza sativa*) is a water-intensive crop, and like other plants uses stomata to balance CO_2_ uptake with water-loss. To identify agronomic traits related to rice stomatal complexes, an anatomical screen of 64 Thai and 100 global rice cultivars was undertaken. Epidermal outgrowths called papillae were identified on the stomatal subsidiary cells of all cultivars. These were also detected on eight other species of the *Oryza* genus but not on the stomata of any other plant species we surveyed. Our rice screen identified two cultivars that had “mega-papillae” that were so large or abundant that their stomatal pores were partially occluded; Kalubala Vee had extra-large papillae, and Dharia had approximately twice the normal number of papillae. These were most accentuated on the flag leaves, but mega-papillae were also detectable on earlier forming leaves. Energy dispersive X-Ray spectrometry revealed that silicon is the major component of stomatal papillae. We studied the potential function(s) of mega-papillae by assessing gas exchange and pathogen infection rates. Under saturating light conditions, mega-papillae bearing cultivars had reduced stomatal conductance and their stomata were slower to close and re-open, but photosynthetic assimilation was not significantly affected. Assessment of an F_3_ hybrid population treated with *Xanthomonas oryzae pv. oryzicola* indicated that subsidiary cell mega-papillae may aid in preventing bacterial leaf streak infection. Our results highlight stomatal mega-papillae as a novel rice trait that influences gas exchange, stomatal dynamics, and defense against stomatal pathogens which we propose could benefit the performance of future rice crops.

## Introduction

Rice is an integral food staple that influences economic and social prosperity (Redfern et al., 2012; Pachauri et al., 2014). It is produced in dry, tropical and temperate climates, on average requiring around 2,500 liters of water to produce 1 kg of grain (Bouman, 2009). With increased temperatures and incidences of severe drought forecast, climate-proofing of rice for future environments will be critical; and as water availability for agriculture is predicted to decrease, there is a focus toward generating rice that is more water-use efficient (Jagadish et al., 2015; Wu et al., 2017; Zhou et al., 2017; Caine et al., 2019; Yang et al., 2019). At the same time, rice breeders need to consider how pathogens such as *Xanthomonas oryzae pv. oryzae* (*Xoo*), which causes bacterial blight, and *Xanthomonas oryzae pv. oryzicola (Xoc*), which causes bacterial leaf streak (BLS), will impact on future yields (Niño-Liu et al., 2006). For *Xoc*, which enters through the same microscopic stomatal pores that govern water-use, yield losses can already exceed 30% (Liu et al., 2014). Despite the potential losses caused by *Xoc*, no studies have investigated how altering stomata on rice leaves might affect *Xoc* infection rate.

For most plants, stomata consist of a pair of guard cells surrounding a central pore (Zeiger et al., 1987). Stomatal opening permits atmospheric CO_2_ uptake for photosynthetic assimilation, and facilitates the diffusion of water vapor in the opposite direction. This loss of water drives a transpiration stream, permitting evaporative cooling and internal movement of solutes from roots to shoots (Hetherington and Woodward, 2003; Hepworth et al., 2015; Caine et al., 2019). Stomatal closure prevents water loss and mitigates against pathogens which enter through the pore - key traits in conserving water and protecting against disease, but it also prevents CO_2_ uptake (Flexas and Medrano, 2002; Martin-StPaul et al., 2017; Ye et al., 2020). Over longer durations, plants can modulate gaseous exchanges and defense against pathogens via changes to stomatal development (Lake et al., 2001; Casson and Gray, 2008; Beerling and Franks, 2009; Dutton et al., 2019). This leads to alterations in the number and or size of stomata that form on developing leaves. By adjusting stomatal opening and stomatal development, uptake of CO_2_ can be tightly balanced with water loss and defense against pathogens. These adjustments to stomatal number, size, and pore aperture help plants to thrive in otherwise adverse environments.

There is considerable morphological variation in stomata between different types of plants, with dicots typically forming pairs of kidney-shaped guard cells and monocot grasses having dumbbell-shaped guard cells (Stebbins and Shah, 1960; Hepworth et al., 2018; Nunes et al., 2020). The stomatal complexes of all grass (and some dicot) species, have subsidiary cells adjacent to their guard cells (Stebbins and Shah, 1960; Rudall et al., 2017). Subsidiary cells are suggested to improve the efficiency of turgor changes in guard cells, thereby enabling quicker opening and closing (Hetherington and Woodward, 2003; Cai et al., 2017; Bertolino et al., 2019). Due to the apparent importance of stomatal complex morphology to grass performance, the volume of associated research is growing rapidly (Liu et al., 2009; Taylor et al., 2012; Chen et al., 2017; Raissig et al., 2017; McKown and Bergmann, 2018; Chatterjee et al., 2020).

Genetic modification (GM) and gene editing have been employed to alter stomatal properties and have demonstrated improved drought tolerance and/or enhanced water-use efficiency in crops and model species (Huang et al., 2009; Yoo et al., 2010; Liu et al., 2012; Hepworth et al., 2015; Hu et al., 2017; Hughes et al., 2017; Li et al., 2017; Caine et al., 2019). This breadth of research demonstrates that there is scope to manipulate stomata to improve plant water-use. However, for many rice producing countries, the growth of crops that have undergone GM or gene editing is currently prohibited (Whitty et al., 2013). Consequently, other naturally derived or non-GM techniques are required to produce plants with stomatal-based changes that improve drought tolerance and or water-use efficiency.

Papillae are dense microscopic outgrowths formed on the epidermis of many plant species including rice (Prasad et al., 2011; Chowdhury et al., 2014; Gorb et al., 2017; Kang et al., 2017; Young et al., 2017). They are found on both earlier and later diverging plant lineages and fulfil a diverse array of functions (Banks and Davis, 1969; Haworth and McElwain, 2008; Duarte-Silva et al., 2013; Chowdhury et al., 2014). In barley (*Hordeum vulgare*) and *Arabidopsis thaliana*, papillae form in response to powdery mildew hyphae, thereby helping to prevent pathogen infection (Thordal-Christensen et al., 1997; Hückelhoven et al., 1999; Maekawa et al., 2014). On lotus leaves, they prevent films of water by reducing the contactable surface area for water droplets, which in turn improves the overall waterproofing of the leaf (Ensikat et al., 2011). In other species, including members of the Proteaceae, papillae aid in photoprotection by reducing the amount of light that passes to the underlying mesophyll (Jordan et al., 2005). Further functions include the production of mucilage and slime in certain lower land plants (Proust et al., 2016), and potentially in regulating gas exchange around stomata (Maricle et al., 2009; Sack and Buckley, 2016).

Reports of papillae developing in close proximity to stomata come from both extant and extinct plant species (Pant and Mehra, 1964; Palmer et al., 1981; Fischer et al., 2010; Prasad et al., 2011). They have been detected on epidermal pavement cells adjacent to stomata and in some instances on subsidiary cells. For grasses, subsidiary cell papillae (SCP) do not appear to be ubiquitous across all members of the Poaceae, instead evidence suggests they are limited to a relatively small number of species, including certain members of the Olyreae (includes bamboo) and Oryzeae tribes (Palmer et al., 1981, 1983; Lima et al., 2020). It has been suggested that papillae forming near stomata may restrict gas flow thereby improving water-use efficiency (Maricle et al., 2009; Lima et al., 2020). However, because papillae have also been detected in species such as rice (Luo et al., 2012; Chatterjee et al., 2020), where water availability is often plentiful, additional roles for papillae are probable. These include preventing water build-up, entry of certain fungal and bacterial pathogens, or protecting against stomatal pore occlusion, such as when volcanic dust particles are prevalent in the atmosphere (Haworth and McElwain, 2008; McElwain and Steinthorsdottir, 2017; Lima et al., 2020). Many molecular components have been detected in papillae including silicon (Si) callose, cellulose, lignins, araboxylans, reactive oxygen species, phenolics, peroxidases, thionins, and aromatic compounds (Thordal-Christensen et al., 1997; Cai et al., 2008; Voigt, 2014; Guerriero et al., 2018).

In this study, we focus on the form and function of abnormally large or abundant papillae, which are located on the subsidiary cells of stomatal complexes of particular rice cultivars, which we term “mega-papillae.” We assess gas exchange, stomatal dynamics and pathogen responses with a view to understanding if mega-papillae could help improve the performance of future rice crops.

## Materials and Methods

### Plant Material and Growth Conditions

The 164 international rice germplasm screened in this study were obtained from either the International Rice Research Institute, Los Banos, Philippines (100 varieties) or the Thai seedbank, Kamphaeng Saen, Thailand (64 varieties) and are listed in Supplementary Table 1.

Seedlings were germinated in Petri dishes filled with water and incubated at room temperature (26–27°C) for 4–5 days with 12 h of daylight. Plants were then transferred to trays filled with clay soil (collected from the field) and were subsequently planted into rice paddies at the Kamphaeng Saen Campus, Nakhon Pathom, Thailand. Screening for stomatal properties of flag leaves was conducted on plants grown under field conditions during February to June 2016. The Dharia (Bangladesh) and Kalubala Vee (Sri Lanka) varieties were selected for further study due to their mega-papillae phenotype. The high yielding IR64 rice variety (IRRI, Philippines) was used as a control due to it having an average size and number of SCP. For observations of papillae on different leaves, seedlings were germinated in Petri dishes filled with ∼20 ml of water and cultivated for 7–8 days in a Sanyo growth cabinet set to 12 h 26°C: 12 h 24°C light: dark, with 200 μmol m^–2^ s^1^ photosynthetically active radiation (PAR). Seedlings were then transferred to 13 cm diameter pots (0.88 L) using the soil mixture described by Caine et al. (2019) and propagated in growth cabinets (Conviron Controlled Environments Ltd, Winnipeg, MB, Canada) at 12 h 30°C: 12 h 24°C light: dark cycle, PAR 1,000 μmol m^–2^s^–1^ and 60% relative humidity. Pots were constantly standing in water, and soil was also supplied with water from above once a week. Plants used for gas exchange and pathogen experiments were grown in 4 liter pots in a greenhouse at Khampaeng Saen Campus with approximately 12 h daily sunlight at ∼348 μmol m^–2^ s^1^ PAR and an average 34°C temperature and 74% humidity, and kept well-watered throughout. Gas exchange experiments were performed on plants which were 75–85 days old, and pathogen experiments were performed on flag leaves of 75 day old plants.

### Sample Collection and Imaging

To observe SCP phenotypes in DH, KV and IR across different leaves, epidermal impressions were taken from leaf 5 (20 to 25 days after germination), maximum tillering (45–50 days after germination) and flag leaf stages (75–80 days after germination) from plants grown in growth chambers. Stomatal impressions from mature leaves of *Carica papaya, Cocos nucifera, Echinochloa crus-galli, Musa acuminate, Oryza officinalis, Oryza rufipogon, Oryza nivara, Oryza puntata, Oryza latifolia, Oryza australiansis, Oryza brachyantha, Oryza ridleyi, and Zea Mays* were collected from plants growing around the Kamphaeng Saen campus between December 2018 to February 2019. For *Arabidopsis thaliana*, *Brachypodium distachyon, Hordeum vulgare, Physcomitrium patens, Osmunda regalis*, and *Selaginella kraussiana* stomatal images were collected from mature leaves or sporophytes grown under controlled conditions.

For quantification purposes, 8 biological replicates per genotype per rice leaf stage were collected. Impressions were taken using dental resin (Coltene Whaledent, Switzerland) on the abaxial surface of leaves, 3/4 of the up way from where the leaf emerged from the sheaf. Nail varnish was applied to the set resin to make imprints for microscopy analysis. Imaging of SCP number and size was conducted using a light compound microscope (Leica, DM750-ICC50 HD), with quantification performed using ImageJ (Schindelin et al., 2012). SCP number was counted from five randomly selected stomata per field of view (FOV) and averaged, with all individual papilla areas on each stomate measured using the Polygon plug-in tool. Measured areas were averaged to give an overall value per stomate, and then the 5 stomate values were averaged to give an average papilla area per FOV. We measured 6 FOVs per replicate per developmental stage.

Scanning electron microscopy samples were fixed in 2.5% Glutaraldehyde for 1 h and washed in 0.1 M Sodium-Potassium phosphate buffer solution for 10 min. Samples were then treated in 1% Osmium tetroxide for 1 h at 4°C, followed by a dehydration series in ethanol concentrations of 30, 50, 70, 80, and 90% for 10 min each, and then rinsed three times further with 100% ethanol for 10 min. To completely dry samples, each sample was placed in critical point dryer. Samples were mounted on aluminum stubs, attached with carbon sticky tabs, and coated with approximately 20 nm of gold via an ion coater (Eiko engineering IB-2). Visualization was performed using a Hitachi SU8020 at an accelerating voltage of 10 Kv. For confocal imaging, samples were collected from fully expanded flag leaves, cut into 3–4 mm strips, then fixed and cleared in modified Carnoy’s solution (acetic acid 7:1 ethanol). Samples were then stained with propidium iodide (PI, 1:100 of a 1 mg/ml stock) for ≥ 5 min and then mounted in chloral hydrate for imaging. For cross-sections, samples were fixed in formaldehyde fixative solution for 2 h and vacuum infiltrated for 1 h. Samples were washed with PEM buffer before dehydration with an ethanol series of 20, 30, 50, 70, 80, 90, 100, and 100% for 30 min each and followed by infiltration with a series of resin in ethanol solutions with resin concentrations of 10, 20, 30, 50, 70, 80, 90, 100, 100, and 100%. The samples were then transferred to gelatin capsules to solidify. Resin capsules were sectioned using a microtome and the sections were stained with 0.25% calcofluor white and washed with 1x PBS before preparing for imaging. Imaging was performed using a Nikon A1 (Tokyo, Japan).

For elemental analysis of papillae, the samples were collected from fully expanded flag leaves and prepared as described for SEM imaging. Chemical content analysis was performed with a Tescan-Mira3 field emission-scanning electron microscope (Czech Republic), with an energy dispersive X-Ray spectrometer (EDS) installed.

### Leaf Gas Exchange

Measurements of *A* and *g*_*s*_ were conducted on fully expanded flag leaves using a LI-COR LI-6400XT Portable Photosynthesis System (Lincoln, NB, United States). Chamber flow rate was set to 400 μmol s^–1^, leaf temperature to 32°C, reference [CO_2_] to 400 ppm and light intensity to 2,000 μmol m^–2^ s^–1^ PAR. Relative humidity inside the chamber was kept at 65–75% using self-indicating desiccant. For light-dark-light response curves, plants were first acclimatized at saturating light (2,000 μmol m^–2^ s^–1^ PAR) for 15–20 min, measurements were then recorded at steady-state for 10 min, this was then followed by 10 min of complete darkness (0 μmol m^–2^s^–1^ PAR), and finally, saturating light was re-applied for the final 10 min. At each light intensity, 20 measurements for *A* and *g*_*s*_ were taken, and intrinsic water use efficiency (iWUE) was calculated as *A*/*g*_*s*_.

### Infection of Rice Hybrids With *Xanthomonas oryzae* pv. *oryzicola*

For pathogen experiments, mega-papillae bearing Dharia was first crossed with Pathum Thani 1 (PT1), a local rice variety commonly grown in Thailand that has a normal number of SCP. F_1_ seeds were left to self-pollinate to produce the F_2_ generation, which was then phenotyped and genotyped to confirm the presence or absence of the mega-papillae trait by using the Kompetitive Allele Specific PCR (KASP) marker and these plants were left to self-pollinate. F_2_ plants were also PCR genotyped to identify those without the *xa5* resistance allele. In the F_3_ generation, 88 seeds from an F_2_ homozygous plant (Papillae phenotype without *xa5*) were grown in trays in the greenhouse and irrigated frequently. To prepare the cell suspension, cultures of *Xanthomonas oryzae* pv. *oryzicola* were grown in a peptone sucrose agar medium and incubated for 48–72 h at 28°C. The bacterial cell suspension was prepared, and the concentration was determined and adjusted to an OD of 0.4 at 600 nm (10^8^ CFU/ml) using a spectrophotometer. Pathogen inoculation was conducted using a spraying method and inoculated plants were kept in the greenhouse without watering for one night, before watering normally again the following day. Disease symptom evaluations were performed 14 days after inoculation with *Xoc*. Disease scoring standard was adapted from the standard evaluation system of the International Rice Research Institute (IRRI, 2013) which corresponds to 5 index values:

1. Index value 1: A resistant plant that almost no symptoms with leaves that are 0–1% infected.
2. Index value 3: A plant that has good resistance with leaves showing greater than 1% but less than 5% infection.
3. Index value 5: A plant that has moderate resistance, which has leaves that are 6–25% infected.
4. Index value 7: A plant that is susceptible with leaves that are 25–50% infected.
5. Index value 9: A very susceptible plant with more than 50% of the leaf being infected.

### Graphs and Statistical Analysis

**FIGURE 1:**
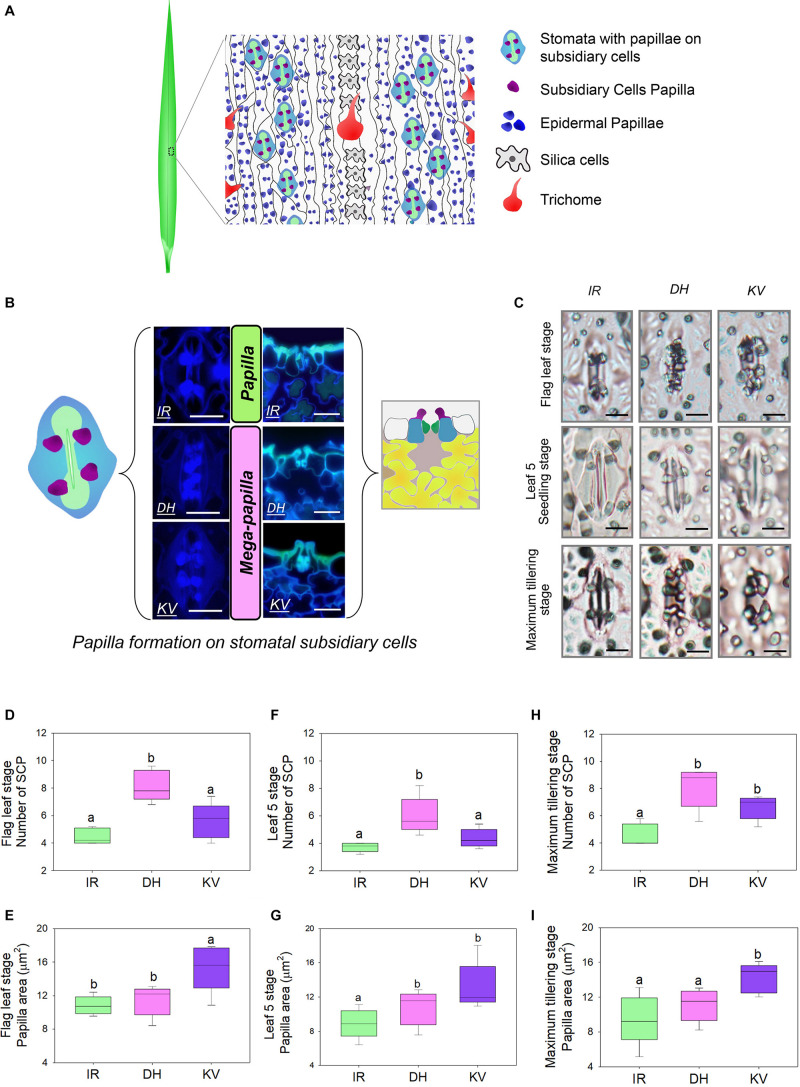
Identification of mega-papillae on the subsidiary cells of stomatal complexes of rice cultivars Dharia (DH) and Kalubala Vee (KV). **(A)** Schematic diagram of flag leaf epidermis illustrating stomatal complexes (guard cells, green; subsidiary cells, light blue) with subsidiary cell papillae (SCP, purple), epidermal cell papillae (dark blue), silica cells (light gray), trichomes and epidermal pavement cells (white with black borders). **(B)** Images of stomatal complexes and SCP from IR64 (IR), Dharia (DH), and Kalubala Vee (KV), taken from above the epidermis via confocal microscopy (left) and taken of the transverse cross sections via fluorescence light microscopy (right) with corresponding schematics. SCP are more numerous for DH, or larger in area for KV, and thus are collectively referred to as mega-papillae. Color scheme as in **(A)**, with underlying mesophyll cells marked in yellow and sub-stomatal cavities in pale pink. **(C)** Light microscopy images of stomatal complexes and SCP of IR, DH, and KV taken from flag leaves (75–80 days), leaf 5 (20–25 days) and at maximum tillering stage (45–50 days). **(D–I)** Number and total area of SCP per stomatal complex of flag leaves **(D,E)**, leaf 5 **(F,G)** and at maximum tillering stage **(H,I)**. For graphs **(D–I)**, horizontal lines within boxes show the median, and boxes the upper (75%) and lower (25%) quartiles. Whiskers indicate the ranges of the minimum and maximum values and different letters indicate values with significantly different means (*P* < 0.05, one-way ANOVA). *n* = 6 plants in **(D–I)**. Scale bar = 10 μm in **(B,C)**.

All graphs were developed, and statistical analysis conducted using Sigmaplot V14 (Systat Software, Inc., San Jose, CA, United States). One-way ANOVAs were performed to determine if there were interactions between samples or treatments for a given parameter measured. *Post hoc* tests (Holm-Sidak) were performed for all graphs on Figures 1, 3B,C,F, 4D,E to identify significant differences between samples.

## Results

### Characterization of Papillae on 164 Rice Cultivars

To identify morphological traits that might aid in improving rice abiotic and biotic stress responses, we examined the abaxial flag leaf surfaces of 164 rice cultivars (Supplementary Table 1) using light microscopy to inspect leaf impressions. Epidermal pavement cells and stomatal complexes were evident, as were silica cells, trichomes and large numbers of small papillae (Figure 1A). For all rice varieties, we found that papillae were not only located on epidermal pavement cells but also on the subsidiary cells of stomatal complexes. There were usually four subsidiary cell papillae (SCP) present on each stomatal complex, with two on each subsidiary cell (see schematic example in Figure 1A). However, for two cultivars, Dharia (DH, *Oryza sativa* L. ssp. *Indica*) and Kalubala Vee (KV, *Oryza sativa* L. ssp. *Indica*), we detected striking SCP phenotypes. Compared to the 162 other cultivars examined, including IR64 (IR, a high-yielding representative example), DH had a greater number of SCP (usually 8 SCP per stomatal complex), and KV had much larger SCP (Figure 1B). The extended shape and/or spatial positioning of SCP on DH and KV, resulted in papillae extending markedly across the guard cells and into the stomatal pore region (Figure 1C). Due to the observed SCP differences, we broadly term the papillae found on DH and KV subsidiary cells as “mega-papillae.”

### Mega-Papillae Are Present on Leaves at Seedling, Maximum Tillering and Flag Leaf Stages

To assess whether mega-papillae occur on the subsidiary cells of expanded leaves prior to the flag leaf stage, we quantified the SCP number and size in DH and KV, and also in IR (for comparison with a cultivar possessing the normal size and number of SCP) across three different growth stages. These were the seedling (leaf 5, 20–25 days old), maximum tillering (45–50 days old), and flag leaf (75–80 days old) stages. To first confirm our initial screening observations, flag leaf SCP were quantitatively assessed (Figures 1D,E). We found that DH had approximately 82% more SCP than IR and 31% more SCP than KV (DH vs. IR, *P* < 0.001; DH vs. KV, *P* < 0.01; Figure 1D), and that KV had approximately 40% larger papillar area compared to IR and DH (IR, *P* < 0.05; DH, *P* < 0.05; Figure 1E). At the leaf 5 stage, we also identified mega-papillae, with DH having significantly higher numbers of SCP than IR and KV (IR, *P* < 0.05; KV *P* < 0.05; Figure 1F) and KV having significantly larger SCP compared to IR and DH (IR, *P* < 0.05; DH, *P* < 0.05; Figure 1G). The same trends were also observed on leaves formed during the maximum tillering stage (Figures 1H,I), although a significant difference was not detected for SCP number between DH and KV (Figure 1H). Overall, the results consistently show that DH plants had the most numerous SCP, whereas KV had the largest SCP area. The number of SCP in DH (compare Figures 1D,F,H), and the size of SCP in KV (compare Figures 1E,G,I) both showed trends toward increasing further on later forming leaves. For the SCP of IR, this was not the case, and the number and size of IR SCP remained similar on all leaves examined.

### Characterizing the Chemical Components of Rice Papillae

**FIGURE 2:**
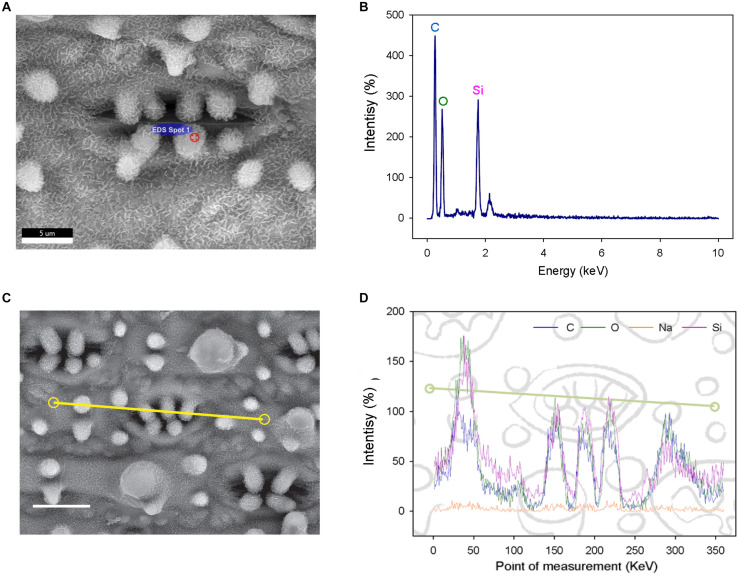
Elemental analysis of subsidiary cell papillae (SCP) and the surrounding epidermal surface on leaves of the Dharia cultivar. **(A)** Scanning electron micrograph (SEM) of a stomatal complex, with a spot measurement of the SCP composition calculated using an energy dispersive X-Ray spectrometer (EDS). **(B)** Element spectrum of an SCP EDS-spot measurement showing all content measured. **(C)** SEM of a stomatal complex illustrating the EDS track path across the epidermal surface (yellow line). **(D)** SEM-EDS spectra of the total of 360 measurement points. Four components were detectable; silicon, oxygen, carbon and a low level of sodium. The peaks represent the concentration of each element at a given point on the line. Scale bars: 5 μm **(A)** and 10 μm **(C)**.

To determine the composition of rice papillae, we assessed the flag leaves of DH (high SCP number) using a scanning electron microscope (SEM) equipped with an energy dispersive X-Ray spectrometer (EDS) (Figure 2). The EDS assessment was conducted using two approaches; (1) spot measurements - which focused solely on stomatal complex papillae (Figures 2A,B); and (2) line scans - which focused more broadly, assessing stomatal papillae and the surrounding stomatal complex and epidermal pavement cells (Figures 2C,D). EDS spot measurements revealed that the DH mega-papillae consisted of four major elements: carbon, oxygen, sodium and silicon (Si). After carbon, Si was the next most abundant element, suggesting that Si is a major component of SCP (Figures 2A,B). EDS line scans provided similar results, with non-stomatal epidermal papillae exhibiting similar elemental signatures to the mega-papillae found on DH stomatal subsidiary cells, with Si again being an abundant element (Figures 2C,D). Several studies implicate Si as being important in pathogen defense (Song et al., 2016; Wang et al., 2017; Mücke et al., 2019), and this could be related to the abundance of silicon in mega-papillae.

**FIGURE 3:**
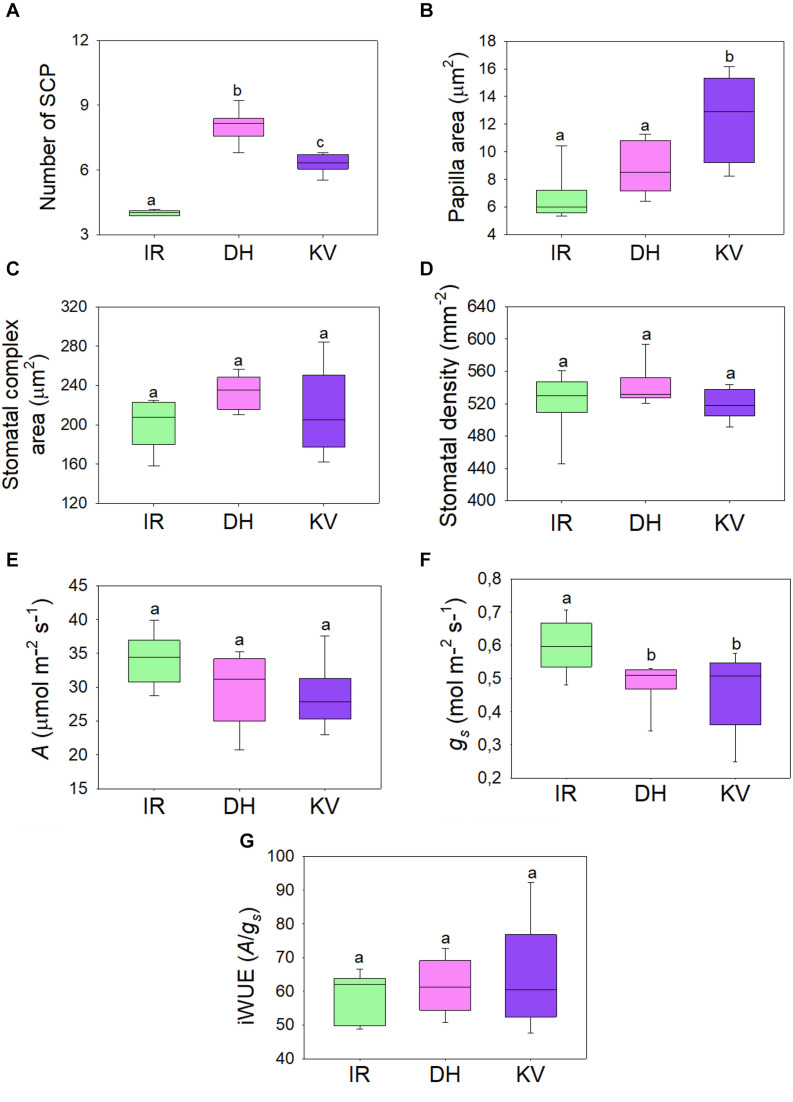
Steady-state gas exchange analysis of mega-papillae cultivars. **(A)** Number and **(B)** total area of subsidiary cell papillae (SCP) per stomatal complex for rice cultivars IR64 (IR), Dharia (DH) and Kalubala Vee (KV). **(C)** Corresponding stomatal complex area and **(D)** stomatal density. **(E–G)** Infra-red gas exchange measurements of **(E)** photosynthetic carbon assimilation rate (*A*), stomatal conductance and the calculated intrinsic water use efficiency (iWUE; *A*/*g*_*s*_). Horizontal lines within boxes show the median and boxes the upper (75%) and lower (25%) quartiles. Whiskers indicate the ranges of the minimum and maximum values and different letters indicate values with a significantly different mean within graph (*P* < 0.05, one-way ANOVA). *n* = 8 plants. Papillae area generated by measuring the outline of each SCP from above view the stomatal complex.

**FIGURE 4:**
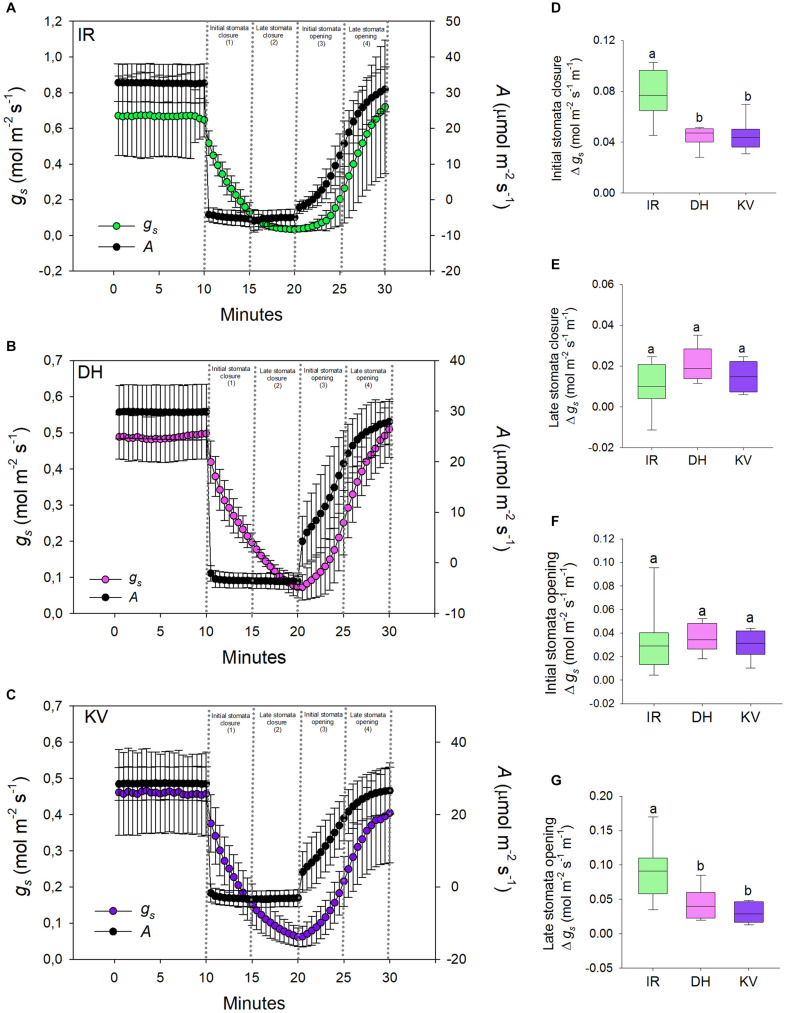
Stomatal responses to dynamic light-dark-light treatments. Flag leaves of **(A)** IR64 (IR), **(B)** Dharia (DH) and **(C)** Kalubala Vee (KV) cultivars treated with 10 min of high light (2,000 μmol.m^− 2^s^− 1^ PAR), followed by 10 min darkness (0 μmol.m^− 2^s^− 1^ PAR), and subsequently another 10 min of high light. The y-axes of graphs show stomatal conductance on the left and photosynthetic carbon assimilation (*A*) on the right. Analysis of *g*_*s*_ rate change per minute during **(D)** initial stomatal closure, **(E)** late stomatal closure, initial stomatal opening and late stomatal opening. For graphs **(A–C)** error bars = one SD. For **(D–G)**, horizontal lines within boxes show the median, with boxes illustrating the upper (75%) and lower (25%) quartiles. Whiskers indicate the ranges of the minimum and maximum values, and different letters indicate values with a significantly different mean within each graph (*P* < 0.05, one-way ANOVA). *n* = 8 plants for DH and KV, and *n* = 7 plants for IR.

### Steady-State Gas Exchange of Mega-Papillae Rice Varieties

To assess whether the gas exchange properties of DH or KV were different to IR, we took infrared gas analyzer (IRGA) measurements from greenhouse-grown plants (Figure 3). SCP number and size were comparable between these greenhouse-grown plants and the growth chamber-grown plants assessed above (compare Figures 1D,E to Figures 3A,B). We also assessed the stomatal complex size and stomatal density of IR, DH, and KV (Figures 3C,D) and found no differences between the 3 cultivars. Under saturating light (2,000 μmol.m^–2^s^–1^ PAR), photosynthetic carbon assimilation (*A*), stomatal conductance and corresponding intrinsic water-use efficiency (iWUE) were measured (Figures 3E,G). Both mega-papillae bearing cultivars, DH and KV, had significantly reduced *g*_*s*_ compared to IR (*P* < 0.05). There were no significant differences in *A* between cultivars (although there appeared to be a trend toward a reduction in *A* in DH and KV), and despite the approx. 20% reduction in *g*_*s*_, no significant differences in iWUE were detected (Figures 3E,G). The lack of difference in iWUE between cultivars could be explained by a reduced *g*_*s*_ influencing the performance of *A*, but we cannot also rule out other factors such as slight differences in photochemical properties between cultivars.

### Mega-Papillae Cultivars Have Stomata That Are Slower to Close and Re-open

Having ascertained that mega-papillae bearing cultivars have reduced steady-state *g*_*s*_ under saturating light, we next investigated the dynamic stomatal performance of DH, KV and IR (Figure 4). To do this, we assessed the light responsiveness of stomata using a light-dark-light illumination treatment. Plants were equilibrated to high light (2,000 μmol.m^–2^s^–1^ PAR) for 15–20 min and then measured at steady-state for 10 min, the light was then turned off for 10 min, and finally light was re-introduced (2,000 μmol.m^–2^s^–1^ PAR) for a further 10 min (Figures 4A–C). As expected, the 10 min dark treatment caused a rapid drop in *A* for all three rice cultivars, whereas the *g*_*s*_ responses were slower. When light was re-applied, *A* was again quicker to respond than *g*_*s*_ in all three cultivars (Figures 4A–C). To explore the efficiency of stomatal opening and closing responses, we calculated *g*_*s*_ rate changes over four 5-min periods (two dark segments and two light segments) (Figures 4D–G). Our data highlighted clear differences in the rate of stomatal responses between IR and the two mega-papillae cultivars during both darkness-induced stomatal closure and during light-induced stomata re-opening. During two of the four time periods, the initial stomatal closure (Figure 4D) and the later stomatal opening (Figure 4G), DH and KV had significantly lower rates of change in *g*_*s*_ compared with IR (*P* < 0.05). *g*_*s*_ decreased over 70% faster in IR than in DH or KV during the first 5 min of darkness, and *g*_*s*_ increased over 60% faster in the 5–10 min following the re-exposure to light in IR compared with DH or KV (Figures 4D,G). As there were no significant differences in stomatal size or density between cultivars (Figures 3C,D), this suggests that the presence of mega-papillae could be associated with the reduced stomatal dynamics observed in DH and KV.

To further assess the dynamic stomatal changes in *g*_*s*_ in Figure 4, we also utilized analytical models that predict *g*_*s*_ response to a single step change in PPFD (McAusland et al., 2016; Vialet-Chabrand et al., 2017). However, because DH and KV had slower closure responses and may not have reached steady-state after 10 min dark-induced stomatal closure, we could not confidently extract time constant values (time to reach 63% of the variation in *g*_*s*_) preventing exact modeled comparisons to be made. Nonetheless, this modeling analysis, shown in Supplementary Figure 1, did add further support to the notion that DH and KV mega-papillae prevent stomata from closing efficiently over shorter time periods (compare Figures 4A,D with Figures 4B,C,E,F), and suggest that further experiments where light fluctuations are altered over a longer duration could be informative in assessing the impact of mega-papillae on stomatal dynamics, *g*_*s*_ and iWUE. Having ascertained that mega-papillae most probably serve as silicon-rich obstacles affecting stomatal dynamics, we next turned our attention to whether mega-papillae can aid in preventing pathogen attack.

### Mega-Papillae Hybrid Plants Have Reduced Bacterial Leaf Streak Symptoms

**FIGURE 5:**
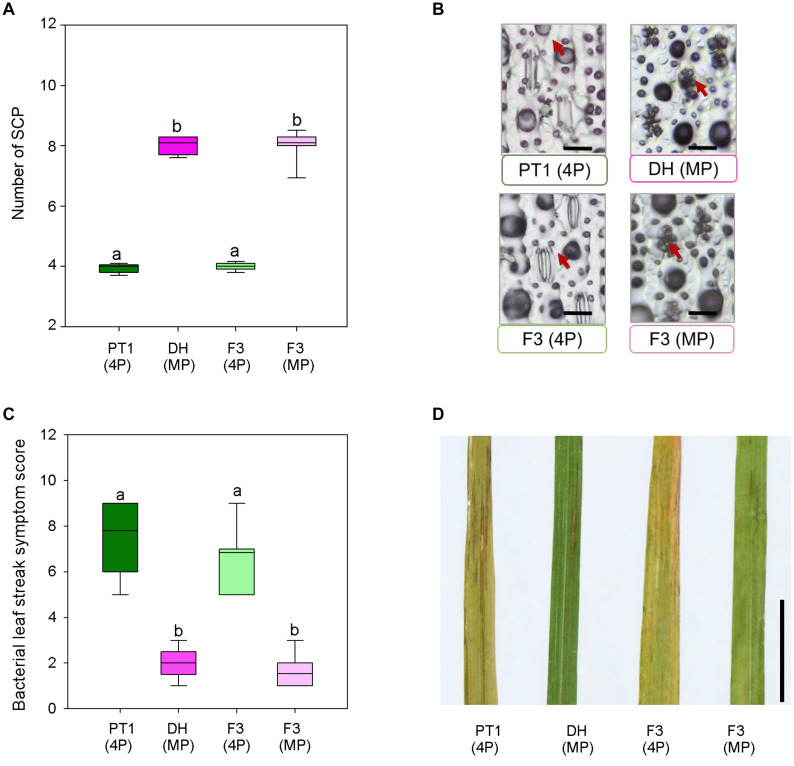
Bacterial leaf streak symptoms of F_3_ hybrid plants generated from four papillae (4P) cultivar Pathum Thani 1 (PT1) and the mega-papillae (MP) cultivar Dharia (DH). **(A)** Number of subsidiary cell papillae (SCP) per stomatal complex of PT1, DH, and F_3_ (4P) and F_3_ hybrid plants. **(B)** Representative images illustrating phenotypes quantified in **(A)**. **(C)** Quantification of bacterial leaf streak symptom scores of plants inoculated 2 weeks prior with *Xanthomonas Oryzae*pv. *oryzicola* (*Xoc*). **(D)** Representative image of leaves assessed to compile the data represented in **(C)**. Scale bars: 50 μm **(B)**, 1 cm **(D)**.

Stomata serve as entry portals for a number of diseases including *Xoc*, which causes rice BLS (Niño-Liu et al., 2006). To examine whether mega-papillae might mitigate *Xoc* infection, we generated an F_3_ population from crosses between the Thai cultivar Pathum Thani1 (PT1), which has the normal number of four SCP per stomatal complex, and the mega-papillae DH cultivar, which unusually has eight SCP per stomatal complex (Figure 5). We also tried crossing DH and KV with IR, but after multiple attempts, we were unsuccessful. To avoid the bias of *xa5* and other QTLs associated with BLS resistance in rice (Sattayachiti et al., 2020), which are present in DH (Iyer and McCouch, 2004) but not in PT1, we used F_3_ individuals segregating for the mega-papillae trait that were derived from F_2_ individuals where the *xa5* and other QTLs were absent (Supplementary Figure 2). From a pool of 88 F_3_ individuals, we selected 26 plants with “normal” SCP and 26 plants with mega-papillae to test for pathogen resistance (Figures 5A,B). We sprayed *Xoc* bacterial suspensions directly on to the mature flag leaves of the identified F_3_ plants during the reproductive stage (75 days old). BLS symptoms were scored using a standard disease scoring scale, and it was evident that F_3_ plants with mega-papillae were approximately 77% less susceptible to *Xoc* than F_3_ plants with the normal number of four papillae (Figures 5C,D). The results for hybrid plants were similar to those of the parental cultivars where DH had approximately 74% less severe disease symptoms than PT1 at 2 weeks after infection (Figures 5C,D).

### SCP Prevalence in the Orzyeae Tribe and Beyond

**FIGURE 6:**
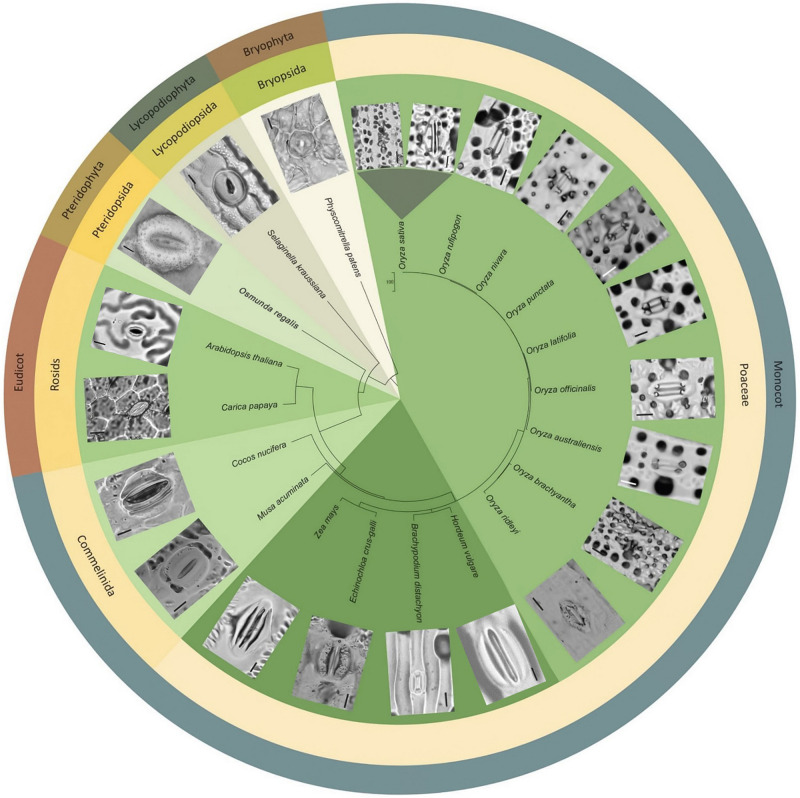
Morphological diversity of stomata and presence or absence of papillae across selected land plant taxa, focusing particularly on *Oryza* species representatives. Stomatal images taken from dental resin impressions or fresh samples using bright-field microscopy. Rice species (*Oryza*) all have subsidiary cell papillae (SCP) on stomatal complexes, whereas other species surveyed do not. Note: papillae are also found on subsidiary cells in bamboo (Palmer et al., 1981; Lima et al., 2020). The associated phylogenetic tree was produced using http://www.timetree.org/ (Kumar et al., 2017). Scale bar = 10 μm.

To assess whether papillae form on the stomatal complexes of other plant types, we next surveyed a range of land plant species across evolutionary clades (Figure 6). Analysis of earlier diverging land plants (bryophtyes and non-flowering vascular plants) and later diverging plants (extant dicots) provided no evidence of papillae on the stomata of any of the species surveyed (Figure 6). We next focused specifically on monocots, first concentrating on some non-grass species (*Cocos nucifera*, coconut and *Musa acuminata*, banana). Despite the presence of subsidiary cells, we did not detect any papillae on or around the stomata of these monocots. Assessment of the true grass species *Echinochloa crus-Galli* (Cockspur grass), *Brachypodium distachyon* (Brachypodium) and *Zea mays* (maize) led to the detection of papillae on the epidermis, but no SCP were present on stomatal complexes (Figure 6). We then looked at other *Oryza* species members to see whether the SCP trait was common in rice species other than *O. sativa*. SCP were present in all the rice species surveyed, including *O. rufipogon, O. officinalis, O. nivara, O. punctata, O. latifolia, O. australiansis, O. brachyantha*, and *O. ridleyi*. Most of these rice species had a similar number and size of SCP to that found in IR. However, two additional SCP traits were identified; in *O. ridleyi*, the SCP appeared to be smaller in size and in *O. brachyantha*, ectopic papillae formed which were not on subsidiary cells. For *O. brachyantha*, papillae arched over the stomatal complex from neighboring epidermal cells. Our results indicate that papillae may well be present, on or adjacent to stomatal complexes, across all rice species but are absent or rare on the stomata of other plant groups.

## Discussion

Plant stomata regulate gaseous exchange with the environment, and in some cases prevent pathogens from entering the leaf interior. In the short term, these processes can be regulated via alterations to stomatal pore aperture, and over longer durations, via changes to stomatal size and/or density (Zeiger et al., 1987; Lake and Woodward, 2008; Martin-StPaul et al., 2017; Dutton et al., 2019; Ye et al., 2020). A number of other specialized epidermal adaptions have also evolved that influence airflow and/or pathogen entry in and around stomata, and these include papillae outgrowths (Barthlott et al., 1998; Hückelhoven et al., 1999; Wakte et al., 2007; Mohammadian et al., 2009; Ensikat et al., 2011; Müller et al., 2017). To find beneficial stomatal or other epidermal traits that might lead to improved rice performance, we screened the flag leaves of 164 different rice varieties. We detected two mega-papillae bearing rice varieties; DH from Bangladesh that has more numerous SCP; and KV from Sri Lanka that has larger S. There have been no previous studies looking at how SCP might affect rice gas exchange, but for DH, work has been conducted highlighting the presence of the *xa5* gene which aids in preventing bacterial leaf blight (Iyer and McCouch, 2004). Given that mega-papillae cover significant portions of the stomatal pore area in both DH and KV, we decided to characterize SCP further, and to study phenotypic responses associated with stomatal function.

DH and KV mega-papillae are present on both earlier and later developing leaves (Figure 1), implying that mega-papillae could be beneficial throughout the majority of both cultivars’ life cycles. Whilst we did not specifically study early mega-papillae development to detect when the corresponding SCP first formed, others have shown that rice SCP formation begins very early in stomatal development around the time that guard mother cells have just divided near the leaf base (Yoo et al., 2011; Luo et al., 2012). SCP development occurs slightly after neighboring epidermal pavement cell papillae. For both types of papillae (pavement cell and SCP), development is concluded well before leaves protrude from encircling sheaves (Luo et al., 2012). These findings suggest that it might be beneficial for rice leaves to have developed papillae prior to beginning to interact with the surrounding environment.

Prior research sheds some light on the molecular underpinnings governing rice SCP development (Yoo et al., 2011; Xia et al., 2015; Zhou et al., 2016). Using a map based cloning approach, Yoo et al. (2011), identified a ROP protein guanine exchange factor OsROPGEF10 as integral for the correct development of both pavement cell papillae and SCP formation. Mutant *bright green leaf* (*bgl*) rice plants have smooth epidermises with both pavement cells and stomatal complexes devoid of Papillae. This leads to increased leaf reflectance (Yoo et al., 2011), but how this impacts on plant physiology, particularly gas exchange and pathogen resistance, is not known. Whether *OsROPGEF10* expression is altered in DH and KV to orchestrate mega-papillae formation is unknown, but when *OsROPGEF10* was over-expressed to rescue the *bgl* mutant, papillae did not seem to be increasingly profuse or larger, and so this seems unlikely. Xia et al. (2015) identified *OsWS1*, a member of the membrane-bound O-acyl transferase gene family, involved in wax biosynthesis, to also be involved in papillae formation. Over-expression of *OsWS1* leads to increased amounts of wax throughout the epidermis, including on SCP, whereas a reduction in *OsWS1* levels resulted in fewer pavement cells papillae and SCP forming. When water-loss and drought studies were undertaken, *OsWS1* over-expressers performed better than controls, whereas plants with depleted *OsWS1* performed worse (i.e., required more water). The *LESS PRONOUNCED LOBE EPIDERMAL CELL 2* (*LPL2*) and *LPL3* genes encode PIR/SRA1-like and NAP1-like proteins, respectively, and both contribute to SCP formation (Zhou et al., 2016). These homologous components of the functionally conserved SCAR/WAVE complex play important roles in actin organization throughout the epidermis. In mutant *lpl2* and *lpl3* plants, the development of small epidermal papillae and SCP are perturbed. In both mutants, fewer, larger papillae form which lack definition. The activities of each of these genes remains unknown in DH and KV and warrants further investigation to determine whether they might contribute to the observed mega-papillae phenotypes.

Our SEM-EDS observations showed that DH mega-papillae have high Si content (Figure 2), and together with the observations of others, this suggests that all papillae on rice leaves, including SCP, are to a large degree comprised of Si (Cai et al., 2008; Yoo et al., 2011). At the plant level, Si is distributed in multiple organs including within roots and leaves, and it is found in a range of epidermal cells including pavement and subsidiary cells (Kumar et al., 2017). Uptake of Si occurs in the roots of rice via specialized Si transporters, *LowSilicon1* (*OsLsi1)* and *OsLsi2* (Ma et al., 2006, 2007). Following this, Si is translocated to the aboveground tissue, with *OsLsi6* involved in xylem unloading and deposition of Si in the leaf sheaths and leaf blades (Yamaji et al., 2008). Our Si SEM-EDS results correspond with previous studies in bamboo and cucumber where high Si content was also observed, and in these cases, Si improved plant pathogen resistance (Kauss et al., 2003; Motomura et al., 2004). Indeed, Si is increasingly becoming associated with pathogen resistance (Ning et al., 2014; Wang et al., 2017). One recent article found that *Xoo* and *Xoc* encode TALE (transcription activator-like effector) proteins that both downregulate *OsLsi1*, and it is therefore intriguing to speculate that *Xanthomonas* bacteria specifically downregulate silicon uptake to increase the chances of successful pathogen attack (Mücke et al., 2019). Future studies looking at *OsLsi1*, *OsLsi2*, and *OsLsi6* functioning in DH and KV might help to reveal how these varieties (1) produce mega-papillae and (2) ward off infection.

We found that the gas exchange rates and stomatal dynamics of mega-papillae bearing cultivars were different to IR, which has normal SCP (Figures 3, 4). Because smaller stomata have been suggested to be faster (Raven, 2014; Lawson and Vialet-Chabrand, 2019), and because more stomata can achieve a higher *g*_smax_ (Bertolino et al., 2019; Caine et al., 2019), we compared the stomatal size and density of mega-papillae bearing cultivars with IR, but found no differences for either stomatal trait (Figures 3C,D). Steady-state gas exchange revealed that DH and KV have significantly reduced *g*_*s*_ in comparison to IR, but despite displaying a trend toward being reduced, *A* was not significantly different between cultivars and nor was iWUE (Figures 3E,G). To test whether mega-papillae hindered dynamic stomatal movements, we next assayed DH, KV and IR using a light-dark-light treatment with the light set at 2,000 μmol.m^–2^s^–1^ PAR (Figure 4). Our results show that DH and KV both displayed reduced *g*_*s*_ rate changes comparatively to IR during both early stomatal closure (Figure 4D) and late stomatal opening (Figure 4G). We note, however, that stomatal or non-stomatal factors other than SCP might contribute to our observed differences in both the steady-state gas exchange and stomatal dynamic movements. Current research has highlighted the importance of “speedy” stomata in grasses which accounts for their characteristic high iWUE values (McAusland et al., 2016; Raissig et al., 2016; Lawson and Vialet-Chabrand, 2019). Our results suggest that under dynamic light environments, having mega-papillae may result in a lower iWUE during both stomatal closing and re-opening; however, these inefficiencies might well be offset by lower *g*_*s*_ under steady-state conditions. How such plants will perform under future predicted high CO_2_ climates is unclear at this stage, and future experiments, particularly at higher temperature and or under water-deficit conditions are required.

The majority of research relating to papillae functionality is in the area of pathogen defense (Ride and Pearce, 1979; Thordal-Christensen et al., 1997; Murillo et al., 1999; Chowdhury et al., 2014; Maekawa et al., 2014; Ning et al., 2014; Wang et al., 2017). Therefore, we examined the role that mega-papillae might play in rice defense against *Xoc*, a pathogen that enters through stomata and causes BLS disease (Niño-Liu et al., 2006; Figure 5). Analysis of F_3_ generation progeny of crosses between DH and PT1 (which did not carry the *xa5* gene as well as other QTLs) revealed that mega-papillae could contribute to increased resistance to *Xoc* (Figures 5C,D). Whether this result is due to an increased physical barrier around the stomatal pore, and/or because of increased Si presence due to more/larger papillae is unclear. Indeed, both of these factors may contribute toward preventing waterborne bacteria from entering underlying tissues. Such waterproofing could also permit sustained gas exchange when otherwise stomata may typically become filled with water. Further experiments looking at the gas exchange rates and stomatal dynamics of DH, PT1, and hybrid plants treated with XOC would give some indication as to whether such a response occurred in rice with mega-papillae. It would also be useful to look at gene expression in the hybrid plants after pathogen inoculation, either via application directly onto leaves or via infiltration, to reveal if other mechanisms other than papillae presence are also employed to achieve the BLS resistance observed in mega-papillae rice lines.

We observed the stomatal morphology across various taxa including dicots, monocots, non-flowering vascular plants and a bryophyte representative (Figure 6) and as expected found a high degree of size and morphological diversity, with earlier diverging land plants and dicots exhibiting kidney-shaped guard cell pairs (except *Physcomitrium patens*: a moss with unusual undivided single guard cells), and monocots exhibiting dumbbell-shaped guard cell pairs surrounded by subsidiary cells. In our assessment, we did not detect papillae near stomata, except on the subsidiary cells of almost all the rice species we surveyed. On the other grasses surveyed, we did detect epidermal pavement cell papillae, suggesting that this may be an ancestral trait within the Poaceae family. Bamboo representatives (closely related to rice) are also reported to have SCP on stomatal complexes (Lima et al., 2020). Given the relatedness of bamboo and rice species, this suggests that the ancestor of Oryzoideae and Bambusoideae may have had SCP on the stomatal complex. A better understanding of the molecular underpinnings of SCP development might in the future help to reveal whether rice and bamboo SCP could have been evolutionarily conserved, or whether SCP have evolved on multiple occasions.

## Conclusion

Here we identify mega-papillae: unusually large or numerous subsidiary cell papillae that appear to limit accessibility to rice stomatal pores. We show that mega-papillae contain silicon and that mega-papillae bearing cultivars have reduced *g*_*s*_ rate changes during stomatal closing and re-opening. The presence of mega-papillae co-segregates with an increased resistance to *Xoc* infection in hybrid lines suggesting a role for mega-papillae in presenting disease. Future work is required to assess the viability of using mega-papillae to improve future crop performance.

## Data Availability Statement

The original contributions presented in the study are included in the article/Supplementary Material, further inquiries can be directed to the corresponding author/s.

## Author Contributions

MP designed and undertook the first phenotypic screening experiment, conducted the first and second phenotyping of the identified mega-papillae plants, conducted the gas exchange and pathogen experiments, and analyzed the corresponding data. RC helped in designing and conducting the gas exchange experiment, advised in data analysis and helped conceive, and write the manuscript. CH helped in designing and conducting the gas exchange experiments and advised on data analysis and interpretation. EH helped in stomatal imaging and conducting gas exchange experiments. WP contributed to phenotypic measurements and elemental analysis. RB helped in physiological data analysis. SN helped during leaf gas exchange experiments. TT assisted the project and advised on the manuscript concept. SW contributed experimental ideas and helped revise the manuscript. AV, JG, RC, and SA contributed to the original idea of the project and supervised the study and prepared the manuscript. SA conceived the project and provided advice and experimental materials. All authors contributed to the article and approved the submitted version.

## Conflict of Interest

The authors declare that the research was conducted in the absence of any commercial or financial relationships that could be construed as a potential conflict of interest.
