# Supplementary material for: Rice Stomatal Mega-Papillae Restrict Water Loss and Pathogen Entry
Source: Front Plant Sci. 2021 Jun 4;12:677839. doi: 10.3389/fpls.2021.677839 (PMC8213340; doi:10.3389/fpls.2021.677839)
Supplement: Supplementary file 1 [file Data_Sheet_1.docx]

**SUPPLEMENTARY MATERIAL**

**
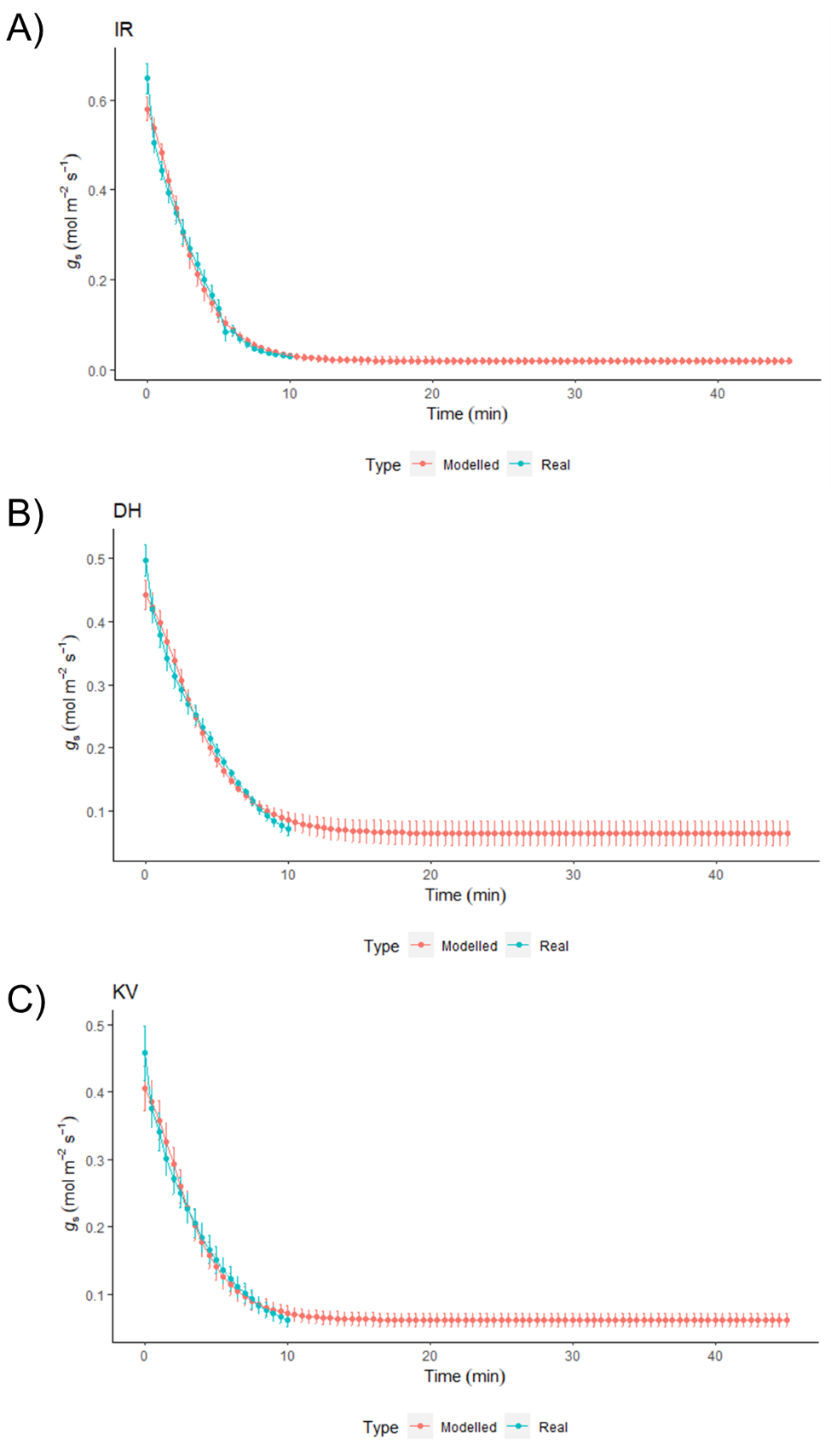
**

**Supplementary Figure 1. Modelled stomatal conductance (*g_s_*) responses to dynamic light change during stomatal closure.** The data fitted in (A) IR64, (B) Dharia, and (C) Kalubala Vee is based on the modeled parameters set out in McAusland et al. (2016) and Vialet-Chabrand et al. (2017). Due to stomatal closure not being complete, particularly in (B) and (C), modelled values to do not tightly fit with actual values, and so exact time constant values (the time to reach 63% of the variation in *g_s_*) cannot be accurately derived. n = 8 plants for DH and KV, and n = 7 plants for IR.


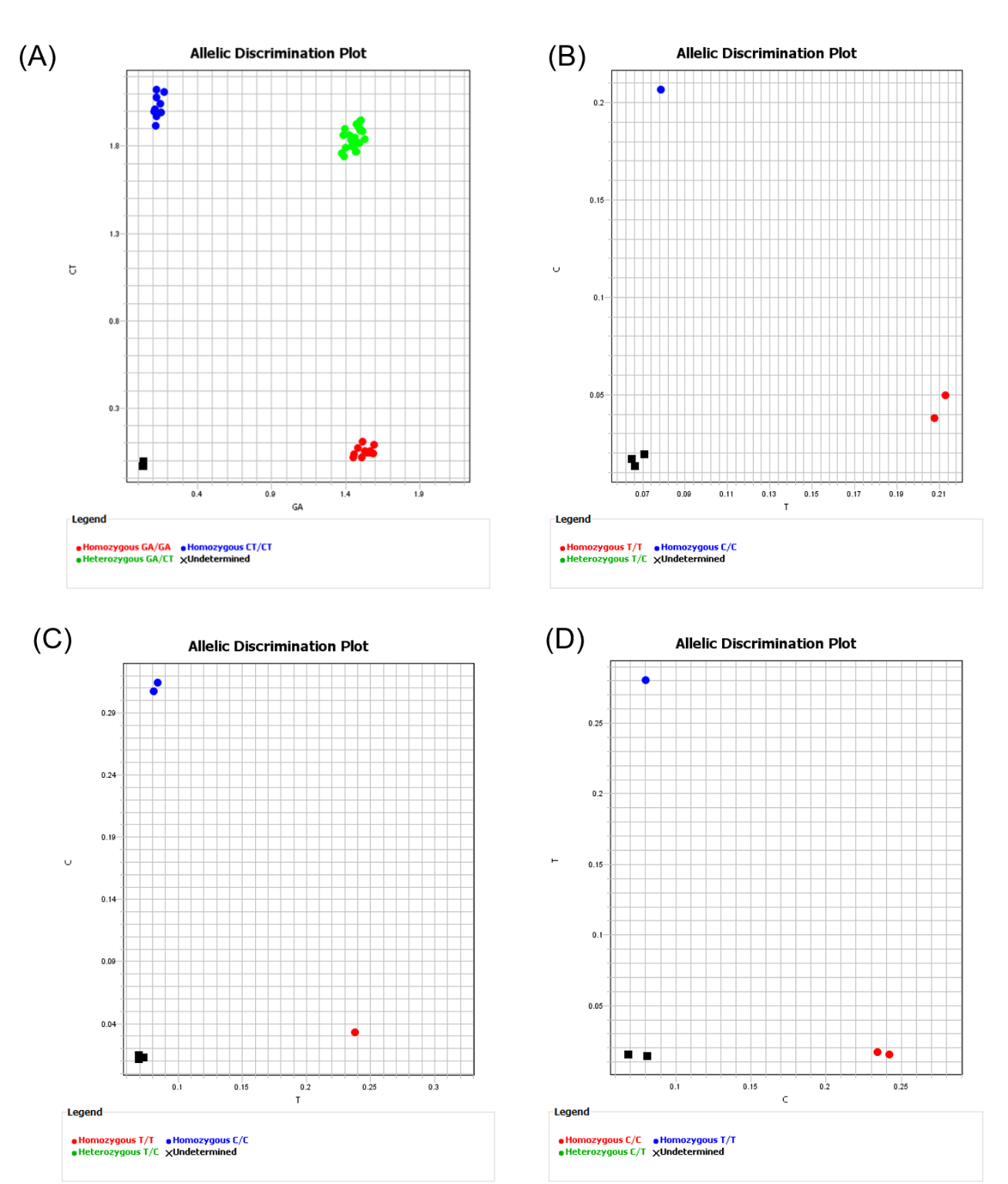


**Supplementary Figure.2 Genotyping of F2 and F3 plants (Dharia x Pathum Thani 1) using markers specific to the bacterial leaf streak (BLS) resistance genes.** Allelic discrimination plot among the 57 individuals of F2 using the marker xa5 (A). Allelic discrimination plot of the sample #103 (F3) using the marker 1_LOC_Os02g331310_2_19708970 (B), 2_LOC_Os02g33180_2_19728136 (C) and 3_LOC_Os02g33230_2_19751892 (D). The sample #103 represents the F3 line with negative alleles (Pathum Thani 1’s alleles) for BLS resistance genes on both chromosomes 2 and 5 (Sattayachiti et al. 2020). The negative alleles for the markers xa5 (A), 1_LOC_Os02g331310_2_19708970 (B) and 3_LOC_Os02g33230_2_19751892 (D) represent by a red dot, and for the marker 2_LOC_Os02g33180_2_19728136 (C) represent by a blue dot.

**Supplementary table 1**

**List of 100 rice line from IRRI collection used in phenotypic screening**

| **No** | **field code** | **Name** | **Country of origin** |
| --- | --- | --- | --- |
| 1 | 1 | B 6136-3-TB-0-1-5 | INDONESIA |
| 2 | 2 | RAY JAZAYKAYZ | BHUTAN |
| 3 | 4 | PURBIA (KALANSAR) | NEPAL |
| 4 | 6 | BALGALA GURMATIA | INDIA |
| 5 | 7 | CAU HAI PHONG | VIETNAM |
| 6 | 8 | NCS741 | INDIA |
| 7 | 9 | 91-385 | BHUTAN |
| 8 | 11 | SINDURI | INDIA |
| 9 | 12 | ERH-CHIANG-TSAO 8 | CHINA |
| 10 | 17 | PADDY 01300 DE MADAG | BURKINA FASO |
| 11 | 32 | GENE | PHILIPPINES |
| 12 | 36 | KHANG KHAOUAY | LAOS |
| 13 | 42 | BJ1 | INDIA |
| 14 | 44 | DA28 | BANGLADESH |
| 15 | 47 | PTB30 | INDIA |
| 16 | 49 | DA8 | BANGLADESH |
| 17 | 50 | KARKATI 87 | BANGLADESH |
| 18 | 52 | KALUBALA VEE | SRI LANKA |
| 19 | 55 | DJ 24 | BANGLADESH |
| 20 | 57 | DZ78 | BANGLADESH |
| 21 | 58 | DK 12 | BANGLADESH |
| 22 | 59 | DD 62 | BANGLADESH |
| 23 | 63 | HEENBALAWEE | SRI LANKA |
| 24 | 65 | ARC 7229 | INDIA |
| 25 | 70 | ARC 10376 | INDIA |
| 26 | 72 | ARC 11959 | INDIA |
| 27 | 78 | GARIA | BANGLADESH |
| 28 | 80 | JABARSHAIL | BANGLADESH |
| 29 | 82 | LAKHSNIKAJAL | BANGLADESH |
| 30 | 83 | MICOCHU | BANGLADESH |
| 31 | 89 | TEPI BORO | BANGLADESH |
| 32 | 90 | KHAO DAWK MALI 105 | THAILAND |
| 33 | 93 | SATHRA 278 | PAKISTAN |
| 34 | 94 | KOALARETA | INDIA |
| 35 | 95 | AUS 55 | BANGLADESH |
| 36 | 96 | AUS 196 | BANGLADESH |
| 37 | 98 | AUS 439 | BANGLADESH |
| 38 | 107 | KELE BARDHAN | INDIA |
| 39 | 108 | SIMUL KHURI | INDIA |
| 40 | 109 | SURJA MUKHI | INDIA |
| 41 | 110 | JAMRI | BANGLADESH |
| 42 | 112 | LALSAITA | INDIA |
| 43 | 114 | CN10183-S/C-244 | JAPAN |
| 44 | 115 | N 22 | INDIA |
| 45 | 116 | T 26 | INDIA |
| 46 | 117 | KALU A. 30 | SRI LANKA |
| 47 | 119 | NCS183 | INDIA |
| 48 | 120 | HEGRA | INDIA |
| 49 | 125 | CHIKON SHONI | BANGLADESH |
| 50 | 126 | DHARIA | BANGLADESH |
| 51 | 127 | DHARIA BOALIA | BANGLADESH |
| 52 | 129 | HOLOI BASH(SOLOI BASH) | BANGLADESH |
| 53 | 160 | SADA DANGA BORO | BANGLADESH |
| 54 | 161 | FR 13 A | BANGLADESH |
| 55 | 163 | 1-GEO-TZE | TAIWAN |
| 56 | 169 | IM16 | BURKINA FASO |
| 57 | 172 | DA9 | MAURITIUS |
| 58 | 174 | NEANG VENG 339 E 23 | BANGLADESH |
| 59 | 175 | 849 | MADAGASCAR |
| 60 | 178 | BRITISH GUIANA 79 | GUYANA |
| 61 | 179 | MAKALIOKA 34 | MADAGASCAR |
| 62 | 181 | PTB25 | INDIA |
| 63 | 182 | B 404 | MYANMAR |
| 64 | 184 | MADAEL | SRI LANKA |
| 65 | 186 | PRATAO | BRAZIL |
| 66 | 187 | RTS4 | VIETNAM |
| 67 | 188 | PADI HOJONG | INDONESIA |
| 68 | 197 | S624(AC 398) | INDIA |
| 69 | 199 | NORUNKAN | SRI LANKA |
| 70 | 206 | JC93 | INDIA |
| 71 | 210 | BPI 76 NON-SENSITIVE(GREEN) | PHILLIPPINES |
| 72 | 211 | TADUKAN | PHILLIPPINES |
| 73 | 217 | PIHATUWEE | SRI LANKA |
| 74 | 218 | KH. NASOUANE | LAOS |
| 75 | 219 | PODIWEE | SRI LANKA |
| 76 | 220 | LUMBINI | SRI LANKA |
| 77 | 224 | HEENDIKWEE | SRI LANKA |
| 78 | 225 | KANNI MURUNGA | SRI LANKA |
| 79 | 227 | VELLAI SEENETTI | SRI LANKA |
| 80 | 230 | ELWEE | SRI LANKA |
| 81 | 237 | PTB1 | INDIA |
| 82 | 238 | DAW LEUANG NAM PUENG 29-3-14 | THAILAND |
| 83 | 241 | PINURSIGI | PHILLIPPINES |
| 84 | 242 | BENZER | PHILLIPPINES |
| 85 | 243 | BG90-2 | SRI LANKA |
| 86 | 247 | CHAMPA TONG 54 | THAILAND |
| 87 | 250 | GIAL HONDERAWAA | SRI LANKA |
| 88 | 251 | GODA HEENATI | SRI LANKA |
| 89 | 253 | LUA SE | VIETNAM |
| 90 | 254 | CARREON | PHILLIPPINES |
| 91 | 269 | JYOT | BANGLADESH |
| 92 | 272 | ARC 15027 | INDIA |
| 93 | 280 | BHASAMANIK | INDIA |
| 94 | 281 | US037 | CAMEROON |
| 95 | 282 | PANAKALI | SRI LANKA |
| 96 | 283 | POONAGARI PERUMAL | SRI LANKA |
| 97 | 284 | UNNAMED | SRI LANKA |
| 98 | 288 | GUAN-YIN-TSAN | CHINA |
| 99 | 290 | T 315 | INDIA |
| 100 | 292 | VATAN | SRI LANKA |

**Supplementary table 2**

**List of 64 Thai-germplasm rice used in phenotypic screening**

| **Line** | **name** |
| --- | --- |
| 1 | GS4000 |
| 2 | GS14386 |
| 3 | GS16233 |
| 4 | GS3816 |
| 5 | GS220 |
| 6 | GS24602 |
| 7 | GS843 |
| 8 | GS4216 |
| 9 | GS6380 |
| 10 | GS6902 |
| 11 | AYUTTHAYA |
| 12 | GS2023 |
| 13 | GS21962 |
| 14 | GS1237 |
| 15 | GS16433 |
| 16 | GS3972 |
| 17 | GS12923 |
| 18 | GS3779 |
| 19 | GS13014 |
| 20 | GS24592 |
| 21 | GS4827 |
| 22 | GS8161 |
| 23 | GS6754 |
| 24 | GS1033 |
| 25 | GS4229 |
| 26 | GS6122 |
| 27 | GS2409 |
| 28 | GS3185 |
| 29 | GS21857 |
| 30 | Banna423 |
| 31 | GS4371 |
| 32 | GS16580 |
| 33 | GS6757 |
| 34 | GS1284 |
| 35 | GS7032 |
| 36 | GS17758 |
| 37 | GS3388 |
| 38 | GS1274 |
| 39 | GS1947 |
| 40 | GS13745 |
| 41 | GS9673 |
| 42 | GS10613 |
| 43 | GS16579 |
| 44 | GS24590 |
| 45 | Sungyod-Pattalung |
| 46 | KaiModRin 3 |
| 47 | GS8226 |
| 48 | GS4446 |
| 49 | GS7419 |
| 50 | GS16239 |
| 51 | GS44525 |
| 52 | GS3092 |
| 53 | GS1281 |
| 54 | GS6302 |
| 55 | GS7065 |
| 56 | GS7130 |
| 57 | GS3767 |
| 58 | GS23436 |
| 59 | GS24607 |
| 60 | GS8137 |
| 61 | GS21963 |
| 62 | GS9821 |
| 63 | GS7032 |
| 64 | GS11191 |

**Supplementary table 3**

**Genotype data of 57 plants of F2 generation from Dharia (Papillae) crossed to Phatum Tani1 (Less papillae).** The F3 population used in pathogen experiment was derived from the sample no 103.

| **No.** | **Sample name** | **Phenotype** | **Genotype** |
| --- | --- | --- | --- |
| 1 | PT 1 | Less papillae | Homozygous GA/GA |
| 2 | Dharia (126) | Papillae | Homozygous CT/CT |
| 3 | 3 | Less papillae | Heterozygous GA/CT |
| 4 | 10 | Less papillae | Homozygous GA/GA |
| 5 | 21 | Less papillae | Homozygous GA/GA |
| 6 | 28 | Less papillae | Heterozygous GA/CT |
| 7 | 72 | Less papillae | Heterozygous GA/CT |
| 8 | 94 | Less papillae | Heterozygous GA/CT |
| 9 | 109 | Less papillae | Homozygous GA/GA |
| 10 | 113 | Less papillae | Heterozygous GA/CT |
| 11 | 115 | Less papillae | Homozygous GA/GA |
| 12 | 120 | Less papillae | Heterozygous GA/CT |
| 13 | 128 | Less papillae | Heterozygous GA/CT |
| 14 | 134 | Less papillae | Heterozygous GA/CT |
| 15 | 143 | Less papillae | Homozygous GA/GA |
| 16 | 152 | Less papillae | Heterozygous GA/CT |
| 17 | 154 | Less papillae | Heterozygous GA/CT |
| 18 | 161 | Less papillae | Homozygous CT/CT |
| 19 | 167 | Less papillae | Homozygous GA/GA |
| 20 | 171 | Less papillae | Heterozygous GA/CT |
| 21 | 180 | Less papillae | Homozygous GA/GA |
| 22 | 182 | Less papillae | Heterozygous GA/CT |
| 23 | 184 | Less papillae | Heterozygous GA/CT |
| 24 | 202 | Less papillae | Heterozygous GA/CT |
| 25 | 222 | Less papillae | Homozygous CT/CT |
| 26 | 226 | Less papillae | Heterozygous GA/CT |
| 27 | 232 | Less papillae | Homozygous CT/CT |
| 28 | 239 | Less papillae | Homozygous CT/CT |
| 29 | 7 | Papillae | Heterozygous GA/CT |
| 30 | 22 | Papillae | Heterozygous GA/CT |
| 31 | 33 | Papillae | Homozygous GA/GA |
| 32 | 48 | Papillae | Heterozygous GA/CT |
| 33 | 59 | Papillae | Heterozygous GA/CT |
| 34 | 60 | Papillae | Homozygous CT/CT |
| 35 | 84 | Papillae | Heterozygous GA/CT |
| 36 | 85 | Papillae | Heterozygous GA/CT |
| 37 | 87 | Papillae | Homozygous GA/GA |
| 38 | 88 | Papillae | Heterozygous GA/CT |
| 39 | 96 | Papillae | Homozygous GA/GA |
| 40 | 101 | Papillae | Homozygous GA/GA |
| 41 | **103** | **Papillae** | **Homozygous GA/GA** |
| 42 | 106 | Papillae | Heterozygous GA/CT |
| 43 | 116 | Papillae | Homozygous CT/CT |
| 44 | 121 | Papillae | Heterozygous GA/CT |
| 45 | 127 | Papillae | Homozygous GA/GA |
| 46 | 151 | Papillae | Heterozygous GA/CT |
| 47 | 175 | Papillae | Homozygous CT/CT |
| 48 | 177 | Papillae | Heterozygous GA/CT |
| 49 | 178 | Papillae | Heterozygous GA/CT |
| 50 | 204 | Papillae | Homozygous CT/CT |
| 51 | 212 | Papillae | Homozygous CT/CT |
| 52 | 216 | Papillae | Homozygous GA/GA |
| 53 | 217 | Papillae | Heterozygous GA/CT |
| 54 | 241 | Papillae | Heterozygous GA/CT |
| 55 | 243 | Papillae | Homozygous CT/CT |
| 56 | 153 | Papillae, Less papillae | Heterozygous GA/CT |
| 57 | 225 | - | Heterozygous GA/CT |
